# Sociodemographic, clinical, and psychosocial factors associated with burden in older caregivers: a cross-sectional study

**DOI:** 10.1590/1980-5764-DN-2022-0030

**Published:** 2023-04-14

**Authors:** Sofia Cristina Iost Pavarini, Allan Gustavo Bregola, Bruna Moretti Luchesi, Nathália Alves de Oliveira, Ana Carolina Ottaviani

**Affiliations:** 1 Universidade Federal de São Carlos, Departamento de Gerontologia, São Carlos SP, Brazil.; 2 Universidade Federal de São Carlos, Programa de Graduação em Enfermagem, São Carlos SP, Brazil.; 3 Central London Community Healthcare NHS Trust, London, England.; 4 Universidade Federal do Mato Grosso do Sul, Programa de Graduação em Enfermagem,Três Lagoas MS, Brazil.

**Keywords:** Aged, Caregiver Burden, Family Health, Idoso, Fardo do Cuidador, Saúde da Família

## Abstract

The task of caring can negatively affect the physical and mental health; therefore, it is important to understand which factors are associated with burden in older caregivers of older adults.

**Objective:**

This study aimed to explore sociodemographic, clinical, and psychosocial factors associated with burden in older caregivers of older adults.

**Methods:**

This is a cross-sectional study developed with 349 older caregivers who were registered at a Family Health Unit of a city in the state of São Paulo, Brazil. Household interviews were conducted and data were collected on the sociodemographic (profile, family income), clinical (self-reported pain, sleep, frailty), and psychosocial (burden, family functioning, depressive symptoms, stress) characteristics of the caregivers as well as dependence on activities of daily living and cognition in the care recipients.

**Results:**

Women predominated in the sample (76.5%) and mean age was 69.5 years. The mean burden score was 18.06 points, with 47.9% above the cutoff of 16 points, denoting excessive burden. The bivariate model revealed associations between burden and financial insufficiency, family dysfunction, difficulty sleeping, pain, perceived stress, depressive symptoms, frailty, and multimorbidity among the caregivers as well as worse functional and cognitive performance in the care recipients. The controlled model revealed an association between burden and depressive symptoms (β=16.75; 95%CI 1.80–31.68).

**Conclusions:**

We identified an association between burden and depressive symptoms, underscoring the need for the planning and implementation of specific actions directed at caregivers in order to minimize the impact on health and to improve the quality of life.

## RESUMO

A tarefa de cuidar pode afetar negativamente a saúde física e mental, sendo importante compreender quais os fatores associados à sobrecarga em idosos cuidadores de idosos.

**Objetivo:**

Explorar os fatores sociodemográficos, clínicos e psicossociais associados à sobrecarga em idosos cuidadores de idosos.

**Métodos:**

Estudo transversal desenvolvido com 349 idosos cuidadores cadastrados nas unidades de Saúde da Família de um município do interior paulista. Foram realizadas entrevistas domiciliares e coletados dados sobre as informações sociodemográficas (perfil, renda familiar), clínicas (dor autorrelatada, sono, fragilidade) e psicossociais (sobrecarga, funcionalidade familiar, sintomas depressivos, estresse) do cuidador, bem como dados de funcionalidade e cognição dos receptores de cuidados.

**Resultados:**

Houve prevalência do sexo feminino (76,5%), com média de idade de 69,5 anos. A média do escore da sobrecarga foi 18,06 pontos, com 47,9% dos idosos cuidadores acima da nota de corte de 16 pontos. O modelo univariado revelou associação entre sobrecarga e percepção de insuficiência financeira, disfunção familiar, dificuldade de dormir, dor, estresse percebido, sintomas depressivos, fragilidade e multimorbidade, além de pior desempenho funcional e cognitivo dos idosos receptores de cuidados. No modelo controlado houve associação entre sobrecarga e sintomas de depressão (β=16,75; intervalo de confiança — IC95% 1,80–31,68).

**Conclusão:**

Identificamos associação entre sobrecarga e sintomas depressivos, de modo que é necessário planejar e implementar cuidados específicos a fim de minimizar o impacto na saúde dos cuidadores e favorecer a qualidade de vida.

## INTRODUCTION

The aging of the population has culminated in an increase in longevity but has also resulted in an increased need for providing care to older adults. Such care, especially in the home setting, is mainly offered by informal caregivers and family members who provide varied types of care without pay^1^. Most of these informal caregivers are women (wife or daughter) who reside with the care recipient, provide care without assistance, and have no training in caregiving^1,2^.

A change in this profile has been seen in recent years, with an increase in the number of informal caregivers who are also older adults^3,4^. Providing care for an older adult has both positive and negative consequences for the caregiver. The positive aspects include a sense of satisfaction, retribution, a stronger bond and exchange of affection as well as personal and spiritual growth^5^. The negative consequences can be both physical and psycho-emotional, such as pain, an increase in the use of medications, depressive symptoms, stress, and excessive burden related to the care provided^6–8^. These factors can be even more intense when the caregiver is an older adult who also has limitations related to aging, which can affect the caregiver’s health and have negative consequences for the older care recipient^7,8^.

Caregiver burden is a concept that encompasses consequences associated with the care provided. Such burden is related to diverse financial, physical, psychological, and social dimensions in the life of the caregiver and is generally assessed based on subjective perceptions^3,9^. To date, most research on measures of caregiving burden has been quantitative, providing tools that are easily adapted within clinical settings^10^ and valuable information for evidence-based intervention programs. Review studies^11,12^ indicate that the tool most widely employed for evaluating burden was the Zarit Burden Interview (ZBI), in particular the 22-item version. In addition to its psychometric properties, the ZBI has been widely used across languages and cultures^11^.

The literature reports factors associated with caregiver burden. A systematic review identified that caregiver burden is negatively associated with perceived social support in caregivers^13^. A clinical review listed risk factors of caregiver burden, such as the female sex, a low level of schooling, living with the care recipient, providing care more hours of the day, depression, social isolation, financial problems, and not choosing to be a caregiver^14^. The findings of a systematic review suggest the presence of sex and gender differences in caregiving burden, with female caregivers experiencing greater burden compared to their male counterparts^15^.

Among community-dwelling older caregivers, predictive factors of burden are related to the caregiver themselves, such as age, self-rated health, income, and the duration of care^16^. In caregivers of patients with dementia, burden is also related to the characteristics of the patient, such as behavioral and psychological symptoms, and factors related to the caregiver, such as income, sex, schooling, residing with the patient, psychological health, well-being, and symptoms of depression and anxiety^17^. One study showed that pain is normally underestimated in older caregivers and is related to the emotional and physical dimensions of caregiver burden^18^.

The studies cited above were conducted with caregivers of different ages (normally in the adult phase), which impedes the identification of the specificities of older caregivers. Moreover, most studies on caregiver burden are limited to groups of caregivers of patients with specific adverse conditions, such as dementia, cancer, and stroke, which limits the generalization of the data and identification of factors common to diverse care contexts^14^. Thus, there is a need to investigate factors related to caregiver burden in older adults who provide care for dependent older adults. The aim of the present study was to explore sociodemographic, clinical, and psychosocial factors associated with burden in older caregivers of older adults.

## METHODS

A quantitative cross-sectional study selected participants based on the following inclusion criteria: age of 60 years or older, registration at a Family Health Unit (primary care modality) in a city in the state of São Paulo, and providing care to a dependent older adult residing in the same home. To be considered dependent, the care recipients needed to require assistance on at least one basic activity of daily living (BADL) listed in the Katz Index^19^ and/or instrumental activity of daily living (IADL) listed on the Lawton & Brody Scale^20^. These measures were also administered to the older caregivers, who needed to be more independent of the care recipients with whom they lived.

The exclusion criteria were as follows: both older adults independent regarding the performance of BADL and IADL, caregivers with severe hearing or visual impairment that would compromise their ability to answer the questionnaires, communications difficulties the impeded the understanding of the questions, the death of one of the older people in the home, a change of address, and individuals who were not encountered after three attempts on different days and at different times.

The sample was selected from a total of 594 residences listed by the Family Health teams where two or more older adults resided. Among these residences, one of the older adults had deceased in 26 homes, a change of address had occurred in 28 cases, the older people were not encountered after three attempts at 69 homes, the older adults declined to participate in the study in 84 homes, and all older adults were independent regarding the performance of BADL and IADL in 36 homes. Among the remaining 351 residences with 351 older caregivers who answered the questionnaire, 2 were excluded from the present analysis for not having completed the evaluations. Thus, the final sample was composed of 349 older caregivers of dependent older care recipients.

Data collection was performed in the homes of the participants after previous contact by trained researchers between April and November 2014. The interviews were conducted in a single session and lasted approximately 1.5 h. All ethical procedures for research involving human subjects were respected in accordance with Resolution 466/2012 of the National Board of Health. This study was authorized by the municipal Secretary of Health and received approval from the Human Research Ethics Committee of *Universidade Federal de São Carlos* (CAAE: 45904621.7.0000.5504). The statement of informed consent was read and explained to each volunteer and signed by all participants prior to data collection.

The variables of interest were investigated using the following measures:

- Sociodemographic, health-related, and care-related characteristics: Data collected using a questionnaire created by the research team addressing sex (male or female), age (years), income (using the national monthly minimum wage in the first semester of 2014 [R$ 724] as reference), income sufficiency (yes or no), difficulty sleeping (yes or no), self-reported diseases (number), pain (yes or no), financial support in providing care (yes or no), assistance from a health institution for providing care (yes or no), assistance from a social service (yes or no), emotional support (yes or no), and assistance from a religious group (yes or no).
- Level of dependence of care recipient for BADL: Katz Index – ability to perform activities of bathing, dressing, toileting, transferring, continence, and feeding^19^. Care recipients with one or more limitations regarding these activities were considered dependent.
- Level of dependence of care recipient for IADL: Lawton & Brody Scale – degree of dependence regarding performance of activities of housekeeping, handling finances, telephone use, managing medications, mode of transportation, shopping, and preparing meals^20^. For the purposes of analysis, a score of 7 points was considered indicative of complete dependence regarding IADL and a score of 8–20 points was considered indicative of partial dependence.
- Cognition of care recipient: Evaluated using Addenbrooke’s Cognitive Examination – Revised (ACE-R), which employs the Mini-Mental State Examination for the global cognitive assessment and assessment of the following domains: orientation/attention, memory, verbal influence, language, and visuospatial. The final score ranges from 0 to 100 points, with higher scores denoting a better cognitive performance^21^. For the purposes of analysis, a score lower than 65 points was considered indicative of poor cognitive functioning^22^.
- Family functioning: Evaluated using the Family APGAR measure, which is used to analyze satisfaction with Adaptation, Partnership, Growth, Affection, and Resolve. The score ranges from 0 to 20 points, with higher scores denoting better family functioning^23^, a score of 20 points was considered normal family functioning, and scores between 0 and 19 points were considered indicative of some degree of family dysfunction.
- Frailty: Evaluated using unintentional weight loss in the previous year, fatigue considering the previous week, muscle weakness, slow gait, and low physical activity level in comparison to the previous year. Unintentional weight loss, fatigue, and physical activity level were self-reported. Muscle weakness was quantified by the mean of three consecutive measures of grip strength of the dominant hand in kgf using a Jamar hydraulic handgrip dynamometer (Model SH5001, manufactured by SAEHAN^®^, Lafayette, IL, USA), with the result adjusted for sex and body mass index (BMI). Slow gait was assessed by the mean of three measures of the time in seconds required to walk 4.6 m along a straight line on a flat surface at one’s usual pace, permitting the use of a gait assistance device if needed. Based on Fried’s phenotype, three to five components characterized frailty, one or two components characterized pre-frailty, and the absence of components characterized non-frailty^24^.
- Perceived stress: Measured using the Perceived Stress Scale (PSS), which was developed to assess the extent to which individuals perceive their living situation as stressful. The total ranges from 0 to 56 points, with higher scores denoting higher levels of perceived stress^25^. In the present study, a score higher than the median among the participants (17 points) was considered indicative of stress.
- Depressive symptoms: Measured using the Geriatric Depression Scale (GDS-15), which addresses mood in older people. The total ranges from 0 to 15 points, with higher scores denoting a higher level of depressive symptoms^26^. In the present study, a cutoff point of >5 points was considered indicative of depressive symptoms.
- Caregiver burden (dependent variable): Measured using the ZBI, which addresses the perceived impact of providing care on the health of the caregiver. The total is calculated from the sum of the points of the 22 items and ranges from 0 to 88 points, with higher scores denoting a greater intensity of caregiver burden^27^. The older caregivers were divided into two groups based on the median among the participants, with ≥16 points considered indicative of excessive burden.

The data were compiled, entered twice in a blinded manner to the Epidata 3.1 software program, and exported to the Statistical Package for the Social Sciences (SPSS for Windows), version 21 (IBM Inc., Chicago, IL, USA). Sociodemographic, health-related, and care-related data were expressed as absolute frequency (n), relative frequency (%), and mean and standard deviation values (Table 1). Linear regression was used to analyze continuous and categorical independent variables associated with the dependent variable (caregiver burden – continuous variable) (Table 2). Associations with a p-value ≤0.20 in the bivariate analysis were selected and employed using the gradual approach and those with a p-value≤0.05 after adjustments in the multiple analysis remained in the final model. The regression data were expressed as β (beta) values and respective 95% confidence intervals (CI). The adjusted R^2^ of the linear regression was used to analyze the representativeness of the final model regarding the variance in caregiver burden.

**Table 1.** Sociodemographic, clinical, and psychosocial characteristics of older caregivers and dependence and cognition of older care recipients (n=349), São Carlos, Brazil, 2014.

Sociodemographic, clinical, and psychosocial variables	Category	N (%) or mean±standard deviation
	**Older caregiver**	
Sex	Male	83 (23.5)
	Female	267 (76.5)
Age (years)		69.55±7.06
	60–69	202 (57.9)
	70–79	108 (30.9)
	>80	39 (11.2)
Family income (R$)		2316.69 ± 1576.82
	Considered sufficient	167 (47.9)
	Considered insufficient	178 (51.0)
	Not reported	4 (1.1)
Family functioning (APGAR)	Family dysfunction	171 (49.0)
	Normal	176 (50.4)
	Not reported	2 (0.6)
Financial support	No	293 (84)
	Yes	55 (15.8)
	Not reported	1 (0.3)
Emotional support	No	186 (53.3)
	Yes	162 (46.4)
	Not reported	1 (0.3)
Assistance from religious group	No	330 (94.6)
	Yes	18 (5.2)
	Not reported	1 (0.3)
Assistance from health institution	No	205 (58.7)
	Yes	144 (41.3)
Assistance from social service	No	335 (96.0)
	Yes	12 (3.4)
	Not reported	2 (0.6)
Difficulty sleeping	No	182 (52.1)
	Yes	168 (47.9)
Multimorbidity	<3 diseases	68 (19.5)
	>3 diseases	281 (80.5)
Pain	No	133 (38.1)
	Yes	213 (61.0)
	Not reported	3 (0.9)
Perceived stress (PSS)	<17	162 (46.4)
	>17	182 (52.1)
	Not reported	5 (1.4)
Depressive symptoms (GDS)	≤5	270 (77.4)
	>5	75 (21.5)
	Not reported	4 (1.1)
Frailty	Non-frail	81
	Pre-frail	195
	Frail	73
	**Older care recipient**	
BADL (Katz)	Independent	240 (68.8)
	Dependent on 1 or+BADL	109 (31.2)
IADL (Lawton & Brody)	Partial dependence	303 (86.8)
	Total dependence	46 (13.2)
Cognition (ACE-R)		53.85±22.12
	>65	85 (24.4)
	<65	231 (66.2)
	Not reported	33 (9.5)

APGAR: adaptation, partnership, growth, affection, resolve; PSS: perceived stress scale; GDS: geriatric depression scale; BADL: basic activities of daily living; IADL: instrumental activities of daily living; ACE-R: Addenbrooke’s cognitive examination – revised.

**Table 2.** Results of linear regression for factors associated with total score of 22-item Zarit Burden Interview (n=349). São Carlos, Brazil, 2014.

Factors		Bivariate model			Controlled model		
		β	95%CI Lower/upper	p	β	95%CI Lower/upper	p
Sex	Male (ref)	1	–	–	–	–	–
	Female	−0.59	−4.21/3.02	0.747	–	–	–
Age (continuous)		−0.58	−0.27/0.15	0.601	–	–	–
Renda familiar (continuous)		<0.01	<0.01/-0.00	**0.011**	−0.01	<0.01/0.00	0.132
Income considered sufficient	No (ref)	1	–	–	1	–	–
	Yes	−6.65	−9.63/-3.68	**<0.001**	2.40	−3.78/8.60	0.439
Family dysfunction (APGAR)	Absent (ref)	1	–	–	1	–	–
	Present	7.51	4.53/10.49	**<0.001**	5.46	−0.89/11.2	0.074
Financial support for providing care	No (ref)	1	–	–	1	–	–
	Yes	3.47	−0.72/7.67	0.105*	−5.50	−13.5/2.55	0.176
Emotional support for providing care	No (ref)	1	–	–	–	–	–
	Yes	−0.26	−3.35/2.81	0.865	–	–	–
Help from religious group	No (ref)	1	–	–	1		
	Yes	3.96	−2.96/10.89	0.261	–	–	–
Help from health institution	No (ref)	1	–	–	1	–	–
	Yes	2.98	−0.11/6.08	0.059*	4.55	−0.73/9.84	0.090
Help from social service	No (ref)	1	–	–	–	–	–
	Yes	4.18	−4.23/12.60	0.329	–	–	–
Difficulty sleeping	No (ref)	1	–	–	1	–	–
	Yes	4.63	1.60/7.66	**0.003**	1.66	−4.19/7.52	0.570
Stress (PSS)	<17 points (ref)	1	–	–	1	–	–
	>17 points	9.89	6.97/12.81	**<0.001**	2.66	−3.85/9.18	0.416
Multimorbidity	<3 diseases (ref)	1	–	–	1	–	–
	>3 diseases	7.01	3.21/10.81	**<0.001**	−1.56	−7.98/4.85	0.627
Depressive symptoms (GDS)	<5 (ref)	1	–	–	1	–	–
	>5	11.70	8.22/15.18	**<0.001**	16.75	1.80/31.68	**0.029**
Pain	No (ref)	1	–	–	1	–	–
	Yes	4.88	1.78/7.98	**0.002**	4.45	−1.12/10.0	0.115
Frailty	Non-frail (ref)	1	–	–			
	Pre-frail	2.28	−1.17/5.74	0.194*	NA	–	–
	Frail	6.15	1.43/10.86	**0.011**	NA	–	–
BADL (care recipient)	Independent (ref)	1	–	–	1	–	–
	Dependent on <1	8.00	4.80/11.20	**<0.001**	5.18	−1.53/11.89	0.127
IADL (care recipient)	Partially dependent (ref)	1	–	–	1	–	–
	Completely dependent	9.92	5.51/14.33	**<0.001**	10.71	−2.05/23.48	0.098
ACE-R (care recipient)	>65	1	–	–	1	–	–
	<65	3.98	0.47/7.49	0.026	1.71	−4.12/7.56	0.558

APGAR: adaptation, partnership, growth, affection, resolve; PSS: perceived stress scale; GDS: geriatric depression scale; BADL: basic activities of daily living; IADL: instrumental activities of daily living; ACE-R: Addenbrooke’s cognitive examination – revised. *Factors with p<0.2 in bivariate analysis incorporated into controlled model. NA: not analyzed due to insufficient number of participants in categories. Bold indicates statistically significant p-value.

## RESULTS

Data from 349 older caregivers were analyzed. The mean score of the ZBI was 18.06±14.54 points, with 167 (47.9%) individuals above the cutoff of 16 points, denoting excessive burden. Table 1 displays the sociodemographic, clinical, and psychosocial characteristics of the caregivers as well as data on the functional dependence and cognitive status of the care recipients. Mean caregiver age was 69.5 years. Women, a perception of insufficient family income, and the absence of financial and emotional support predominated in the sample of caregivers.

Difficulty sleeping, perceived stress, and depressive symptoms were found in more than half of the caregivers. Reports of pain and multiple morbidities were found in 60 and 80%, respectively. Most care recipients were partially dependent with regard to IADLs and had an ACE-R score indicative of poor cognitive functioning.

Table 2 displays the results of the linear regression analysis for the dependent variable (total score on ZBI). Family income, perception of financial insufficiency, and family dysfunction were social factors associated with a greater perception of burden in the bivariate model. Difficulty sleeping, pain, perceived stress, depressive symptoms, frailty, and multimorbidity were clinical factors that increased the likelihood of a higher score on the burden scale. Poor functional and cognitive performance of the care recipient were associated with an increase in the caregiver burden score. When the factors were controlled in the multiple regression model, only depressive symptoms remained associated with greater caregiver burden.

The adjusted R^2^ revealed that the controlled model explained 32.7% of the variance in caregiver burden. In the bivariate regressions, the perceived stress score (PSS) and depressive symptoms (GDS) each explained 11% of the variance in burden.

## DISCUSSION

This study explored factors related to burden in older adults who provide care for dependent older adults. The bivariate analysis revealed that family income, perception of financial insufficiency, and family dysfunction were sociodemographic factors associated with greater caregiver burden. Difficulty sleeping, pain, perceived stress, depressive symptoms, frailty, and multimorbidity were psychosocial/clinical factors associated with greater caregiver burden. Moreover, poor functional and cognitive performance in the care recipients were factors associated with an increase in caregiver burden. The final regression model revealed that older caregivers with depressive symptoms were 16.75-fold more likely to have a higher score on the ZBI.

The mean burden score was 18.06 points, and 47.9% of the caregivers had a score higher than the cutoff of 16 points. This mean score is comparable to the score reported in the study by James et al.^28^ (16.92±12.04 points), but lower than that reported by Connors et al.^29^ (24.0±15.8 points). However, the studies cited were not conducted exclusively with older caregivers. Divergences among studies may be due to methodological differences and/or sample heterogeneity as well as cultural factors^30^.

The associations between greater perceived burden and family income/perception of financial insufficiency have also been reported in previous studies^31,32^. Care is often provided by a family member, generally a wife, daughter, or daughter-in-law of the dependent older adult. These women make sacrifices in terms of their own personal, professional, and social lives and are commonly not paid or live on the income of the care recipient. Those who receive some type of financial support generally consider the contribution insufficient and those who live on little or no income have fewer options when providing for the needs of the care recipient.^1^ This demonstrates the economic vulnerability of caregivers, which intensifies the degree of stress and burden^31,32^.

Older caregivers in a situation of family dysfunction were more likely to have a higher burden score. Satisfaction with family functioning is closely related to social support, as the support that caregivers receive can be important to coping with stressful situations related to providing care, which can lead to higher levels of burden^33^. Studies have shown that poor quality in terms of social support – whether formal or informal – is associated with a higher level of perceived burden in family caregivers^34,35^. Moreover, good family functioning assists in the maintenance of the health and well-being of older people who provide care for dependent family members^35^.

Regarding clinical and psychosocial characteristics, difficulty sleeping, reports of pain, multimorbidity, perceived stress, depressive symptoms, and frailty were associated with a greater perception of burden. Care-related burden can have long-term negative effects on the physical, emotional, social, and financial state of informal caregivers^3,34,36^. Studies have shown associations between burden and psychological suffering, including stress and depression^2,15^, as well as physical conditions, such as diabetes, hypertension, and arthritis^37^, and a negative impact on subjective well-being^38^. Specifically, depression and a negative impact on physical health are highly prevalent among of caregivers, with an interrupted sleep pattern, pain, and the early transition to frailty syndrome^39,40^.

The occurrence and intensity of these effects on health differ considerably among subgroups of caregivers. Women, married caregivers, and those who provide intensive care seem to have more negative effects on their own health related to providing care^2,36^. Such individuals predominated in the sample of the present study. It is also possible that caregivers neglect their own health, as their health problems may seem less important compared to those of the care recipient^38^. Caregivers may not have enough time or energy to attend appointments at health care services due to the high care demand and absence of support. Moreover, the capacity to provide adequate care to dependent older adults is negatively affected by poor physical and mental health on the part of caregivers^41^.

Poor functional and cognitive performance of the care recipients were external factors associated with the increase in caregiver burden. According to the literature, needs with regard to activities of daily living and low cognitive performance in dependent older people are the strongest predictors of perceived caregiver burden^42,43,44^. Specific conditions, such as dementia and cognitive impairment, also increase the degree of caregiver burden^45^.

A systematic review performed to synthesize determinants of burden among informal caregivers found that longer care duration and a greater degree of dependence of the care recipient were the strongest predictors of greater perceived burden. Besides physical dependence, the mental state of the care recipient in terms of behavioral problems and cognitive capacity were also determinants of the level of dependence and positively related to the degree of caregiver burden^34^.

Depressive symptoms constitute a potential variable to address in interventions directed at caregivers of older people that could have an effect on the subjective perception of care-related burden^46^. A study with caregivers (>18 years of age) of older adults with Alzheimer’s disease found that depressive symptoms were among the variables that exerted a mediating effect on the association between caregiver burden and neuropsychiatric symptoms, such that depressive symptoms may constitute an explanatory variable to understanding the subjective perception of burden^47^. The association between depressive symptoms and caregiver burden in the present study is in agreement with findings reported in studies involving caregivers of older adults in general^19–21,47^ as well as older caregivers of older adults^48^.

The findings of the present study have potential implications for the development of social policies or recommendations to prevent and reduce the occurrence of burden in older caregivers of older adults as well as the establishment of specific interventions, considering risk factors for burden resulting from informal care provided to older adults and variables that can minimize such burden. This is important, as providing care often results in health problems and a reduction in quality of life. However, the results of this study should be considered with caution due to the cross-sectional design and use of a convenience sample, which limit the inference of causality and the generalizability of the results. Moreover, the explanatory capacity of the models used in this study was low to moderate, even after including a substantial number of potentially explanatory variables. This demonstrates the possibility of numerous subjective factors in this process that cannot be quantified or explained using a quantitative approach.
